# Meningococcemia in an 11 Months Old Infant

**DOI:** 10.1155/2023/8951318

**Published:** 2023-03-08

**Authors:** Rachana Shrestha, Saurab Karki, Manoj Khadka, Suhail Sapkota, Bibek Timilsina, Sulochana Khadka

**Affiliations:** ^1^Department of Pediatrics, Shree Birendra Hospital, Kathmandu, Nepal; ^2^Military Hospital, Itahari, Sunsari, Nepal; ^3^Nepalese Army Institute of Health Sciences, Kathmandu, Nepal; ^4^Hospital for Advanced Medicine and Surgery, Kathmandu, Nepal

## Abstract

Meningococcemia is the infection of the blood caused by *Neisseria meningitidis*. Herein, we report a case of meningococcemia in an 11 months old infant who had a high-grade fever, nonblanching purpuric rash over the face and limbs, low blood pressure, tachycardia, and prolonged capillary refill time, but without neck rigidity and focal neurologic signs. He recovered after supportive care and treatment with antibiotics (intravenous ceftriaxone, vancomycin, and teicoplanin). Therefore, in a febrile, ill-looking child in shock with a nonblanching rash, meningococcal disease should be suspected. The study shows the importance of vaccination against meningococcal disease.

## 1. Introduction

Meningococcal disease, caused by *Neisseria meningitidis*, is a rare disease with high morbidity and mortality in children [1]. It should be suspected when an ill-looking febrile child presents with a nonblanching purpuric rash [2, 3]. Meningococcal disease can present as meningitis and meningococcemia, the latter being more dangerous [3].

Since meningococcemia is life-threatening and more dangerous than meningitis, waiting for meningitis to label meningococcal disease might be harmful, so if features of meningococcemia are seen, it should be identified early and managed promptly. Thus, early diagnosis is important, and when meningococcemia is suspected, treatment should start immediately, and otherwise, if either diagnosis or management is delayed, then there is an increased risk of mortality [3, 4].

Herein, we report a rare case of meningococcemia without features of meningitis in an 11 months old infant who recovered after treatment with supportive care and antibiotics. The takeaway message from this case is that when a febrile, ill-looking child in shock presents with a nonblanching purpuric rash without signs of meningitis, clinicians should suspect meningococcemia for early diagnosis and management of the rare and life-threatening case. The study also highlights the importance of meningococcal vaccination to protect against the fatal consequences of the disease and recommends the provision of meningococcal vaccination in the routine immunization schedule.

## 2. Case Presentation

An 11 months male infant, first by birth order from Kathmandu, was brought to Shree Birendra Hospital (SBH), Kathmandu, with a high-grade fever for one day, preceded by a cough and running nose two days back. After fever, the child started developing rashes, first noticed by parents at the dorsum of the right hand, which was pinpoint-sized initially and gradually spread to all limbs and the face. The child was lethargic, refused to suck, and had decreased urine output. He was immunized as per the National Immunization Program of Nepal for his age, but since meningococcal vaccination is not included in the routine immunization schedule of the country, the meningococcal vaccine was not taken. There was no significant past, family, and psychosocial history.

**Figure 1:**
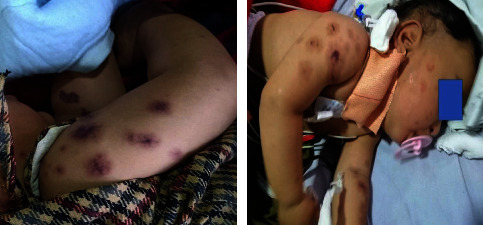
Purpuric rash over the upper limbs and face of an 11 months old infant.

On examination, the child was irritable and lethargic with a purpuric rash over the face and all four limbs. He was poorly responsive with pallor and edema of the hands, and cold hands and feet. Pulse was 130 beats per minute, regular, and low volume with a fever of 101 degrees Fahrenheit. Respiratory rate was 45 breaths per minute. Blood pressure was 90/50 (less than the 50^th^ centile), and capillary refill time was greater than 3 seconds. Oxygen saturation was 98% in room air. Central nervous system (CNS) examination revealed a lethargic child with decreased muscle tone, but there was no neck rigidity and focal neurological signs. Rashes were nonblanching, purpuric with petechiae and ecchymosis over the face and limbs (Figure 1).

Blood parameters revealed a hemoglobin of 8.6 gm% with normal white blood cell (WBC) and platelet count and normal coagulation profiles. C-reactive protein (CRP) was positive while erythrocyte sedimentation rate (ESR) was 46 mm/hour with D-dimer greater than 10,000 ng/ml. Cerebrospinal fluid (CSF) analysis revealed glucose of 59 mg/dl, protein of 61 mg/dl, and cell count of six lymphocytes (Table 1). No organism was seen in the Grams stain and acid-fast bacilli (AFB) stain. Similarly, his blood and urine cultures were sterile.

The child was treated with intravenous (IV) fluids (20 ml/kg normal saline bolus thrice and half-strength normal saline along with 5% dextrose as maintenance fluid), intravenous paracetamol 15 mg/kg/dose when required, antibiotics (intravenous ceftriaxone 100 mg/kg/day 12 hourly, intravenous vancomycin 20 mg/kg/dose 8 hourly, and intravenous teicoplanin 10 mg/kg/dose 12 hourly then 6 mg/kg/dose IV 12 hourly), steroid (intravenous dexamethasone 0.6 mg/kg/day 6 hourly), vasopressors (11 mcg/kg/min injection dopamine and 7 mcg/kg/min injection dobutamine), transfusion of 15 ml/kg packed red blood cells (PRBC) and fresh frozen plasma (FFP) each once, intravenous midazolam 1 mg stat and when needed, and ointment 2% mupirocin 12 hourly. Slowly, the child recovered and was discharged home.

The child was brought to the hospital after 1 week following discharge for follow-up and was playful and cooperative.

## 3. Discussion

Meningococcal disease can present as meningitis (infection of the membrane covering the brain and spinal cord) or meningococcemia (infection of the blood) or a combination of both [3, 5]. It is caused by *Neisseria meningitidis*, a Gram-negative encapsulated diplococcus, present in the human nasopharynx as commensals [5, 6]. It infects humans only and spreads through direct contact with infected nose or throat discharges [5]. When the organism gains access to the systemic circulation, it causes meningococcemia.

Although the disease can occur at any age, it is common in infants due to the loss of maternal antibodies and lack of protective antibodies formation [5–7]. Our case is also an 11 months old infant. A case of meningococcemia reported from Portugal was also an infant [1], and an observational study from the UK showed the median age for meningococcal infection was two years [2]. The disease can be rapidly fatal leading to death within hours, so it should be diagnosed as early as possible [8]. However, due to the rarity of the disease, clinicians see very few cases during their career which pose a diagnostic difficulty [8]. When a nonblanching rash is present along with purpura, deranged capillary refill time, and hypotension in an ill-looking child, then meningococcal infection should be suspected, and further treatment should be commenced in the line of meningococcal infection as soon as possible [2]. Our case also presented these features. An observational study from the UK also showed that children with meningococcal disease were more likely to be ill and have a fever, purpura, hypotension, and prolonged capillary refill time [2]. The classic features of meningitis such as neck stiffness and photophobia usually occur late in the meningococcal disease, and it is hazardous to wait for these symptoms to occur [3, 8].

Bacteriological culture is useful in the diagnosis of meningococcal disease but may have less sensitivity, especially when done after starting antibiotic treatment [6]. The negative culture reported in our case can also be caused by the initiation of antibiotic treatment before doing a culture analysis. A retrospective population-based study in West Gloucestershire showed that out of 252 cases of invasive meningococcal disease, 83% were diagnosed based on culture, whereas 16% were clinically diagnosed, and the remaining 1% were diagnosed by methods other than culture [4]. Thus, clinical diagnosis can be important when the culture comes negative, especially when the antibiotic is started before the culture analysis. Blood culture can be positive in up to 3/4^th^ of cases [6].

Meningococcemia needs to be diagnosed early and managed with antibiotics for treating infection and fluids along with vasopressors for the management of shock [3]. Our case was also managed with antibiotics, fluids, vasopressors, and blood components, which is similar to the case reported in Portugal [1]. Delay in diagnosis or management can increase the risk of mortality [3].

The mainstay to control meningococcal disease is immunization. Antibodies against the capsule of the organism protect from infection, which forms the basis for the vaccination against meningococcal disease [6, 9]. Conjugate meningococcal vaccine and polysaccharide meningococcal vaccine are the currently available vaccines for meningococcal disease [9]. The vaccines licensed for use in infants are 2 doses of a monovalent conjugate meningococcal vaccine, with at least 2 months intervals in between the doses, followed by a booster dose after a year for infants aged 2–11 months, and 3 doses of combined Haemophilus influenzae type B (HIB) plus monovalent C meningococcal vaccine at 2, 4, and 6 months of age followed by a booster at 12–15 months of age [9]. Our case was not immunized with a meningococcal vaccine, highlighting the importance of meningococcal vaccination.

## 4. Conclusion

We report a case of meningococcemia in an 11 months old infant who presented with high-grade fever, nonblanching purpuric rash over the face and limbs, and features of shock but without signs of meningitis. Thus, in a febrile, ill-looking child with a nonblanching purpuric rash in shock without signs of meningitis, meningococcemia should be suspected. Since meningococcal vaccines are the key to protection against meningococcal disease, we recommend meningococcal vaccination be included in the routine immunization schedule of the country. The study highlights the importance of vaccination to protect against meningococcal disease.

## Figures and Tables

**Table 1:** Laboratory findings.

Lab parameters	Lab values
Hemoglobin (gm %)	8.6
Red blood cells (million/mm^3^)	4.6
Total leucocyte count (/mm^3^)	8,400
Differential count (%)	Neutrophil (58), lymphocyte (40), monocyte (1), eosinophil (1)
Platelet (/mm^3^)	350,000
Prothrombin time (seconds)	Test (20.8), control (14.5)
International normalized ratio (INR)	1.4
Activated partial thromboplastin time (seconds)	45.8
D-dimer (ng/ml)	>1,0000
Fibrin degradation products (micrograms/ml)	>150
CSF glucose (mg/dl)	59
CSF protein (mg/dl)	61
CSF cell counts	6 (lymphocytes)

## Data Availability

The data used to support the findings of this study are included within the article.
